# Growth hormone receptor gene disruption in mature‐adult mice improves male insulin sensitivity and extends female lifespan

**DOI:** 10.1111/acel.13506

**Published:** 2021-11-22

**Authors:** Silvana Duran‐Ortiz, Edward O. List, Yuji Ikeno, Jonathan Young, Reetobrata Basu, Stephen Bell, Todd McHugh, Kevin Funk, Samuel Mathes, Yanrong Qian, Prateek Kulkarni, Shoshana Yakar, Darlene E. Berryman, John J. Kopchick

**Affiliations:** ^1^ Edison Biotechnology Institute Ohio University Athens Ohio USA; ^2^ Molecular and Cellular Biology program Ohio University Athens Ohio USA; ^3^ Department of Biological Sciences College of Arts and Sciences Ohio University Athens Ohio USA; ^4^ Barshop Institute for Longevity and Aging Studies San Antonio Texas USA; ^5^ Department of Biomedical Sciences Heritage College of Osteopathic Medicine Ohio University Athens Ohio USA; ^6^ Diabetes Institute Ohio University Athens Ohio USA; ^7^ Department of Molecular Pathobiology David B. Kriser Dental Center New York University College of Dentistry New York New York USA

**Keywords:** aging, Cre‐Lox, growth hormone, IGF‐1, insulin sensitivity, lifespan, tamoxifen

## Abstract

Studies in multiple species indicate that reducing growth hormone (GH) action enhances healthy lifespan. In fact, GH receptor knockout (GHRKO) mice hold the Methuselah prize for the world's longest‐lived laboratory mouse. We previously demonstrated that GHR ablation starting at puberty (1.5 months), improved insulin sensitivity and female lifespan but results in markedly reduced body size. In this study, we investigated the effects of GHR disruption in mature‐adult mice at 6 months old (6mGHRKO). These mice exhibited GH resistance (reduced IGF‐1 and elevated GH serum levels), increased body adiposity, reduced lean mass, and minimal effects on body length. Importantly, 6mGHRKO males have enhanced insulin sensitivity and reduced neoplasms while females exhibited increased median and maximal lifespan. Furthermore, fasting glucose and oxidative damage was reduced in females compared to males irrespective of *Ghr* deletion. Overall, disrupted GH action in adult mice resulted in sexual dimorphic effects suggesting that GH reduction at older ages may have gerotherapeutic effects.

## Graphical abstract

Attenuation of growth hormone action in mice at a mature adult age (6‐months) extends lifespan, reduces protein oxidation and glomerulonephritis in females while improving insulin sensitivity and decreasing lipid peroxidation, glomerulonephritis, and neoplasms in males.

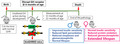

**Abbreviations**
1.5mGHRKO: 1.5‐months GH receptor disrupted mice
6mGHRKO: 6‐months GH receptor disrupted mice
AdGHRKO: adipocyte‐specific growth hormone receptor knockout mice
ALS: acid‐labile subunit
AOiGHD: adult‐onset GH‐deficient mice
AT: adipose tissue
AUC: area under the curve
fPAPP‐A/pos: pregnancy‐associated plasma protein‐A disrupted mice
FSH: follicle stimulating hormone
gas: gastrocnemius
GH: growth hormone
GH−/−: GH knockout mice
GHR: growth hormone receptor
GHRH: GH releasing hormone
GHRKO: GH receptor knockout mice
GTTs: glucose tolerance tests
HNE: 4‐hydroxynonenal
IGF‐1: insulin growth factor‐1
IGFBP‐2: insulin growth factor binding protein‐2
IGFBP‐3: insulin growth factor binding protein‐3
ITP: Interventions Testing Program
ITTs: insulin tolerance tests
JAK2: janus kinase 2
L2‐Cmu: GF‐1R monoclonal antibody
LH: luteinizing hormone
LID: liver–specific IGF‐1
LS: Laron Syndrome
peri: perigonadal
quad: quadriceps
SOCS2: suppressor of cytokine signaling 2
STAT5: Signal transducer and activator of transcription 5
subq: subcutaneous
Tam: tamoxifen
TG: triglycerides

## 1 INTRODUCTION

Growth hormone (GH), promotes growth, inhibits insulin action (diabetogenic effect) (Vijayakumar et al., 2010), stimulates expression of insulin growth factor‐1 (IGF‐1) by the liver and other target tissues (Kopchick & Andry, 2000), and induces both catabolic and anabolic effects in tissue dependent manners (Vijayakumar et al., 2010). Importantly, numerous studies have shown that GH is part of an evolutionarily conserved pathway of genes whose expression modulate the aging process. Studies performed in yeast, worms, fruit flies, and mice show that disruption of GH and/or IGF‐1(or their homologs) can improve health and extend lifespan (Junnila et al., 2013). A link between the GH/IGF‐1 axis and aging has been also shown in humans. That is, a sub population of Ashkenazi Jewish centenarians and their offspring harbor mutations in the IGF‐1 receptor, resulting in decreased activity of the GH/IGF‐1 axis (Suh et al., 2008). Additionally, studies by Guevara‐Aguirre and colleagues reveal that humans with Laron Syndrome (LS) who are GH‐resistant, have enhanced insulin sensitivity, are resistant to diabetes and cancer, and show a significant reduction in pro‐aging markers (Guevara‐Aguirre et al., 2011).

Several mouse lines with germline GH axis disruptions have shown extensions in lifespan. Specifically, mice with inactivating gene mutations in the GH releasing hormone (GHRH) or its receptor (also known as the lit/lit mouse), as well as Ames and Snell mice (congenital mutations in pituitary transcription factors), and the GHR gene disrupted (−/−) or knockout (GHRKO) mice, exhibit decreased body length, increased body adiposity, improved glucose metabolism, and markedly extended lifespan (Bartke, 2008). As a result, it has been proposed that targeted inhibition of the GH axis could be a promising pharmacological intervention to extend healthy aging (Longo et al., 2015). Notably, except for the GHRKO mice, the aforementioned mouse lines have reduced action of at least one additional hormone such as prolactin, thyroid‐stimulating hormone, or GHRH, that may contribute to their extended longevity phenotype. Therefore, the GHRKO mouse line was established as a model to study the specific effects of reduced GH action in vivo. Furthermore, the GHRKO mice are a model for subjects with LS, who also harbor inactivating mutations in the GHR gene and show significant decrease in serum IGF‐1, increased GH levels and have a reduced body size. Importantly, due to their exceptional longevity, the GHRKO mice hold the Methuselah mouse prize for the world's longest‐lived laboratory mouse with a lifespan a week short of 5 years of age (Pilcher, 2003). The GHRKO mice also exhibit improved healthspan, showing improved cognition and insulin sensitivity, resistance to diabetes, reduced neoplasia, and decreased markers of aging such as adipose tissue (AT) senescence (Stout et al., 2014) and mTORC1 signaling in liver, kidney, heart, and muscle (Fang et al., 2018; Stout et al., 2014). Our laboratory recently reported that some of the benefits of congenital GH deficiency, such as enhanced insulin sensitivity and extended lifespan in females could be achieved if GHR is disrupted during puberty at 1.5 months of age (Junnila et al., 2016). In light of such promising results, the present study sought to answer if it is possible to attenuate GH action further in life and attain the benefits obtained in mice with congenital GHR ablation. Clinically relevant interventions to extend healthy lifespan should be given at an adult age. Therefore, here, we disrupted the GHR at 6 months of age in mice (corresponding to ~30 years old in humans) (Hagan, 2020), where the animals have already completed sexual maturity. This study will determine if an intervention given at a mature‐adult age to reduce GH action can enhance health and lifespan.

## 2 RESULTS

### 2.1 Modulation of the GH/IGF‐1 axis following GHR disruption at 6 months Age

**FIGURE 1:**
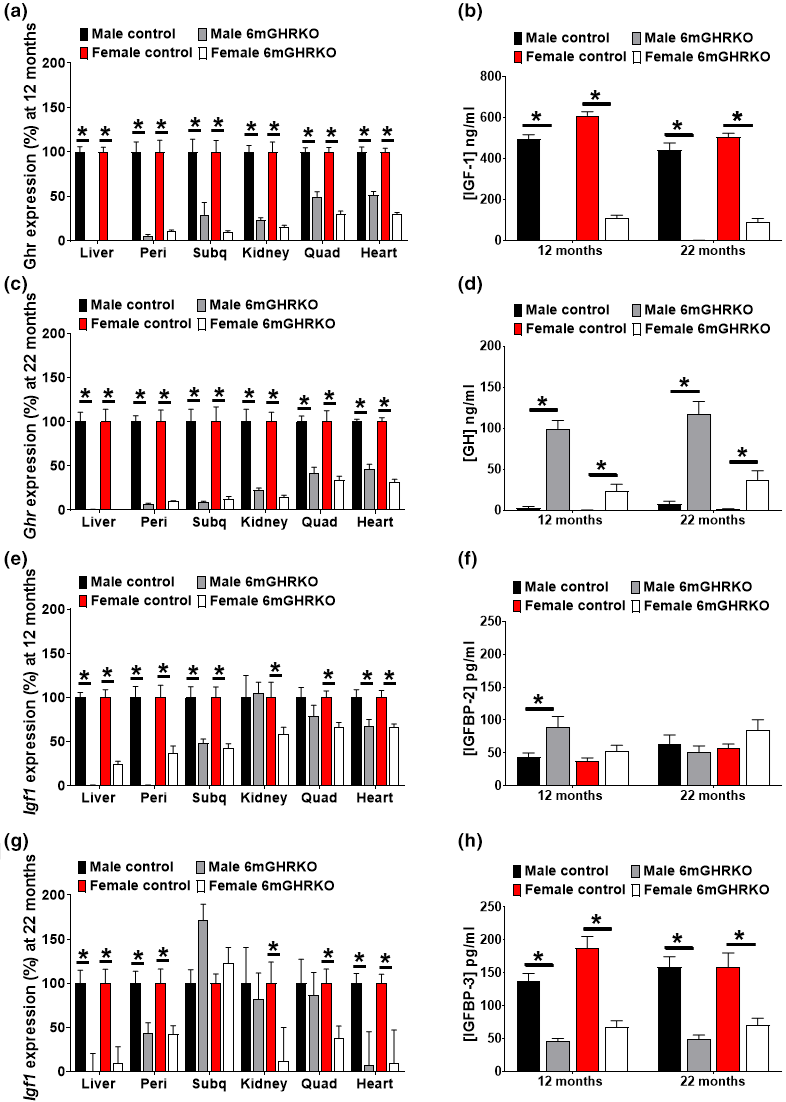
GH/IGF‐1 axis is altered as a result of *Ghr* gene disruption at 6 months of age. (a) *Ghr* gene expression at 12 months of age (n = 12/group). (b) Circulating IGF‐1 levels at 12 and 22 months of age (n = 9/group). (c) *Ghr* gene expression at 22 months of age (n = 13/group). (d) Circulating GH levels at 12 and 22 months of age (n = 9/group). (e) *Igf‐1* gene expression at 12 months of age (n = 12/group). (f) Circulating IGFBP‐2 levels at 12 and 22 months of age (n = 9/group). (g) *Igf‐1* gene expression at 22 months of age (n = 13/group). *Ghr* and *Igf‐1* mRNA levels were determined by RTqPCR. (h) Circulating IGFBP‐3 levels at 12 and 22 months of age (n = 9/group). Black bars represent male and red bars female controls, whereas gray‐ and white bars represent male and female 6mGHRKO mice, respectively. All values are mean ± SE. Student two‐tailed T tests were used to assess differences between 2 groups (female or male 6mGHRKO mice vs. controls). *p ≤ 0.05. Subq, subcutaneous; Peri, perigonadal; Quad, quadriceps

To generate the 6mGHRKO mice, we used the Tamoxifen (Tam)‐induced Cre‐Lox system driven as previously described (Duran‐Ortiz et al., 2018). The efficacy of Tam‐induced gene recombination was assessed by evaluating *Ghr* gene expression using RT‐qPCR in several tissues of mice at middle age (12‐months) and old age (22‐months): liver, kidney, two adipose tissue (AT) depots (subcutaneous [subq] and perigonadal [peri])‐, quadriceps (quad), and heart. *Ghr* gene expression was significantly decreased at both ages in all the tissues in both sexes. Specifically, liver and kidney, showed ~99% and ~80% decrease in *Ghr* expression. Peri and subq AT depots showed ~90% and ~80% reduction in *Ghr* expression, respectively (Figure 1a,c). Skeletal muscle and heart were less responsive to Tam induction, showing at least 48% decrease in *Ghr* expression levels in both sexes at both time points. Together, Tam injection at 6 months of age was sufficient for reduction of *Ghr* gene expression that lasted throughout life.

Growth hormone stimulates 75%–90% of the circulating IGF‐1 production from the liver (List et al., 2014), which, in turn, reduces GH release from the anterior pituitary via a negative feedback loop. Given that *Ghr* gene ablation in the liver was exceptionally effective (>99%), it is not surprising that circulating IGF‐1 levels were significantly reduced in male and female 6mGHRKO mice as compared to controls and at both 12‐ and 22 months of age (*p* < 0.0001; Figure 1b). Due to the lack of the negative feedback loop on GH release, we also found an increase in circulating GH levels in both sexes and at both time points (*p* < 0.0500; Figure 1d). We also found that *Igf‐1* mRNA expression in most tissues followed a similar trend to that seen for the *Ghr* gene expression; that is, *Igf‐1* mRNA levels were most significantly decreased in liver (76%), followed by peri AT (57%) and heart (33%) of male and female 6mGHRKO mice compared to controls at both time points (Figure 1e,g). However, IGF‐1 production was unaffected in the kidney and skeletal muscle of male mice at both time points and in the subq AT depot at 22 months of age for both sexes (Figure 1e,g). The bioavailability of IGF‐1 is highly dependent on the expression levels of IGFBPs. We found that IGFBP‐2 was increased in males at 12 months of age (*p* = 0.0305; Figure 1f) while IGFBP‐3 was significantly decreased in both sexes and at both time points compared to controls (*p* < 0.0001; Figure 1h).

### 2.2 Body size is minimally affected while body composition is altered in 6mGHRKO mice

**FIGURE 2:**
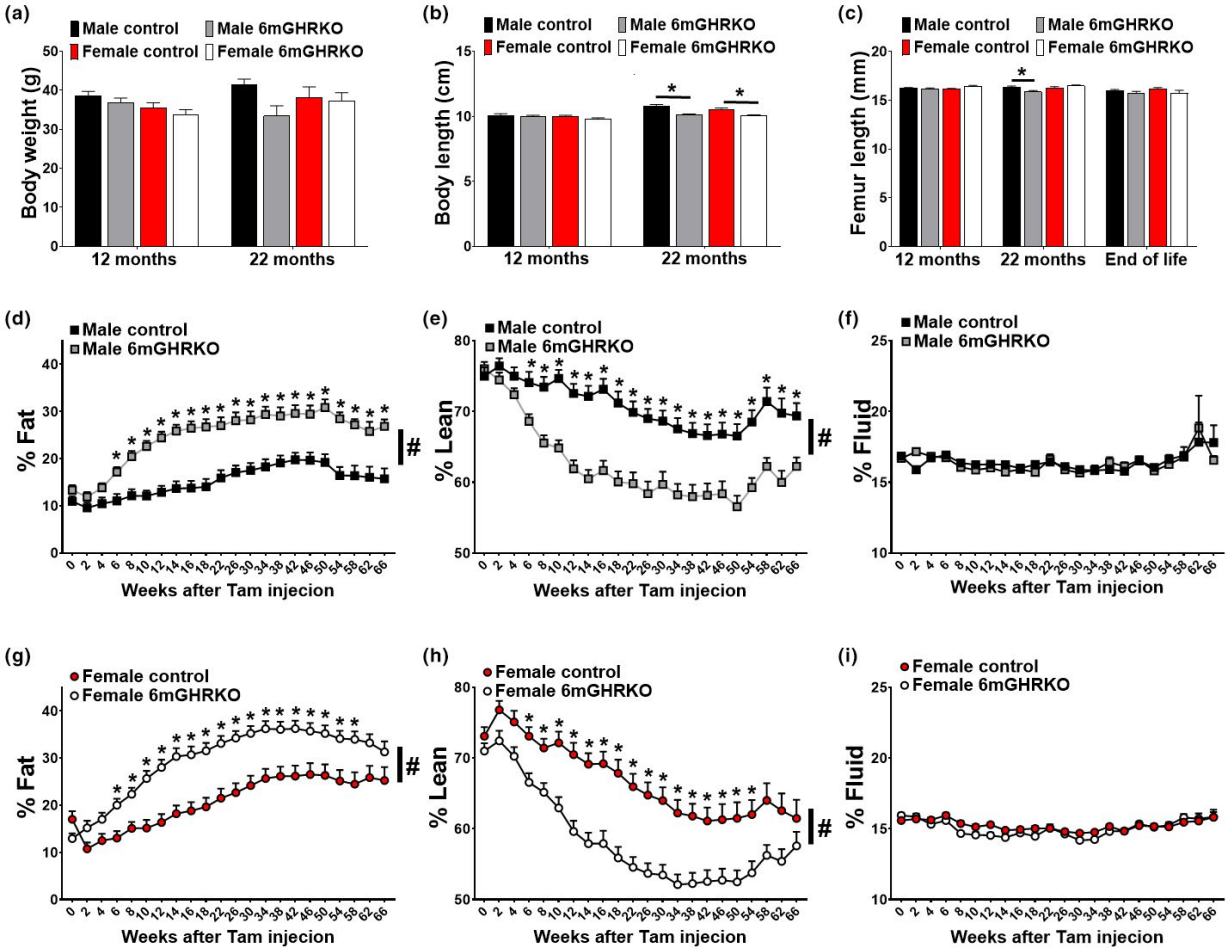
Mice with disrupted GH action at 6 months of age do not show stunted body size but have altered body composition. (a) Bodyweight at the time of dissection 12 (n = 12/group) and 22 (n = 13–14/group) months of age. (b) Body length at the time of dissection (12 and 22 months of age) (c) Femur length at the time of dissection (12 and 22 months of age) and at end of life. (d‐g) Percentage fat mass in males and females over time (n = 13–14/group). (e‐h) Percentage lean mass in males and females over time (n = 13–14/group). (f‐i) Percentage fluid in males and females over time (n = 13–14/group). Black squares and red circles represent controls and gray squares and white circles represent 6mGHRKO mice. Black bars represent male and red bars female controls, whereas gray‐ and white bars represent male and female 6mGHRKO mice, respectively. Student two‐tailed T test was used to assess significant differences between experimental and control mice of the same sex. Repeated measures ANOVA was used for over time assessment and Student T test for difference between individual time points for each sex. All values are mean ± SE. *p ≤ 0.05

Growth hormone has catabolic effects on AT while being anabolic for most other tissues (Vijayakumar et al., 2010). To assess how disruption of GH action in mature‐adult mice alters bodyweight, body composition and body length, we measured these parameters from one day before Tam treatment (6 months old) until the mice were 22 months of age. Repeated measures ANOVA showed that bodyweight over time was not significantly changed in either sex of 6mGHRKO mice compared to controls (Figure 2a and Figure S1). Unlike humans, the bone growth plate of mice does not fuse after sexual maturation (Jilka, 2013). We found that ablation of GHR at a mature‐adult age in male and female 6mGHRKO mice had no impact on body length at 12 months of age, but body length was reduced at 22 months of age with 6mGHRKO males and females showing a mild, but significant reduction of 6.3% and 4.7%, respectively, compared to controls. Furthermore, no significant difference was found in femur length of 6mGHRKO mice at 12 months of age. Although femur length in males at 22 months of age was decreased compared to controls (*p* = 0.0073) (Figure 2b), there were no significant differences in femur length in either sex at the end of life (Figure 2b). Decreased GH action in 6mGHRKO mice significantly altered body composition over time in both sexes compared to controls (Figure 2c‐h), with the percent fat mass increased (*p* < 0.0005) and percent lean mass decreased (*p* < 0.0004). To test whether decreased GH action at a mature‐adult age affects tissue/organ development, we measured total tissue weights, as well as relative tissue weights (tissue/organ weight normalized to bodyweight) at 12 and 22 months of age (Figure S3). We found a sex‐ and age‐dependent reduction in the total and relative weight of most of the tissues (*p* ≤ 0.0500), including internal organs (pancreas, liver, kidney, lung, and intestine) and skeletal muscles (gas and quad). Additionally, the subq AT of male 6mGHRKO mice was consistently increased compared to controls (*p* ≤ 0.0500). Interestingly, the brain was the only tissue that showed opposite results. That is, while an increased brain relative weight was seen in males at 22 months of age (*p* ≤ 0.0001), a significant reduced brain total weight was seen in female 6mGHRKO mice at 12 months of age compared to controls (*p* ≤ 0.0045).

### 2.3 Sex‐specific improvement in insulin sensitivity in 6mGHRKO mice

**FIGURE 3:**
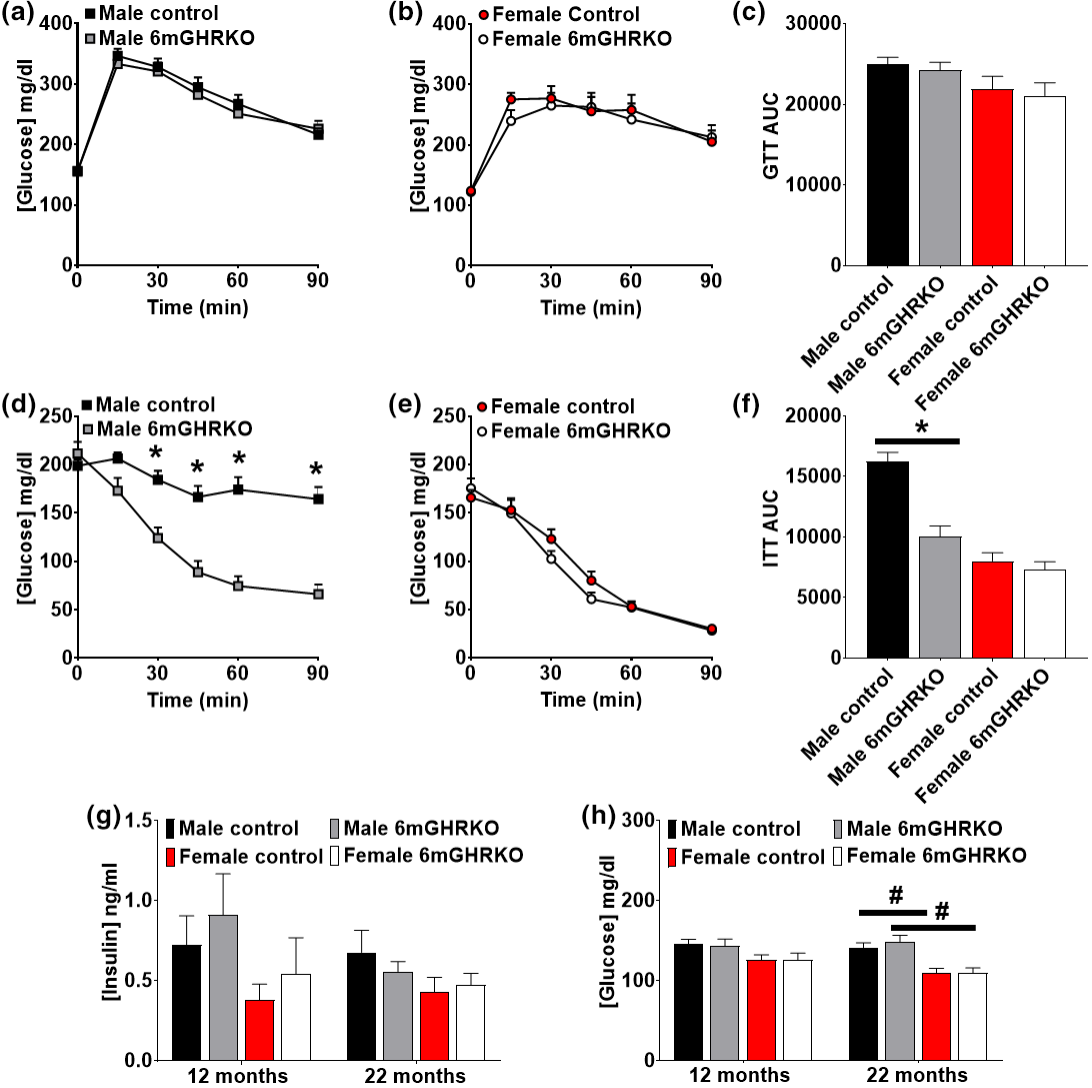
Improved insulin sensitivity in only males and normal glucose tolerance in both males and females 6mGHRKO mice. (a‐b) GTTs in 21‐month‐old male and female 6mGHRKO mice vs. controls (n = 12/group). (c) GTT area under the curve (AUC). (d‐e) ITTs in 21‐month‐old male and female 6mGHRKO mice vs. controls (n = 12/group). (f) ITT area under the curve (AUC) at 21 months of age. (g) Fasting insulin at 12 and 22 months of age. (h) Fasting glucose levels of male and female 6mGHRKO mice vs. controls at 12 and 22 months of age. Black squares and red circles represent controls and gray squares and white circles represent 6mGHRKO mice. Student T test for difference between individual time points for each sex. Black bars and red bars represent male and female controls, whereas gray‐ and white bars represent male and female 6mGHRKO mice, respectively. Student two‐tailed T test was used to assess significant differences between experimental and control mice of the same sex. Two‐way ANOVA was used to evaluate differences between sexes and between experimental groups. All values are mean ± SE. *p ≤ 0.05

Growth hormone's diabetogenic (aka anti‐insulin) effect certainly influences glucose homeostasis (Vijayakumar et al., 2010). Therefore, we performed glucose tolerance tests (GTTs) and insulin tolerance tests (ITTs) at 11‐ and 21 months of age. Glucose tolerance was similar in both sexes at both time points (Figure 3a‐c and Figure S4). Insulin sensitivity, however, was significantly improved only in male 6mGHRKO mice compared to controls at 11‐ and 22 months of age (*p* < 0.0500, Figure 3d,e,f and Figure S4). Fasting insulin levels were not altered 6mGHRKO compared to controls (Figure 3g); to note, the starting fasting glucose levels of the ITT are higher in males compares to females. In fact, at 22 months of age, fasting glucose levels were significantly decreased in females (both control and 6mGHRKO mice) compared to male mice (*p* < 0.0117) (Figure 3h).

### 2.4 Unaltered FSH or LH, liver TG, altered adipokine secretion, and reduced oxidative damage in 6mGHRKO mice

**FIGURE 4:**
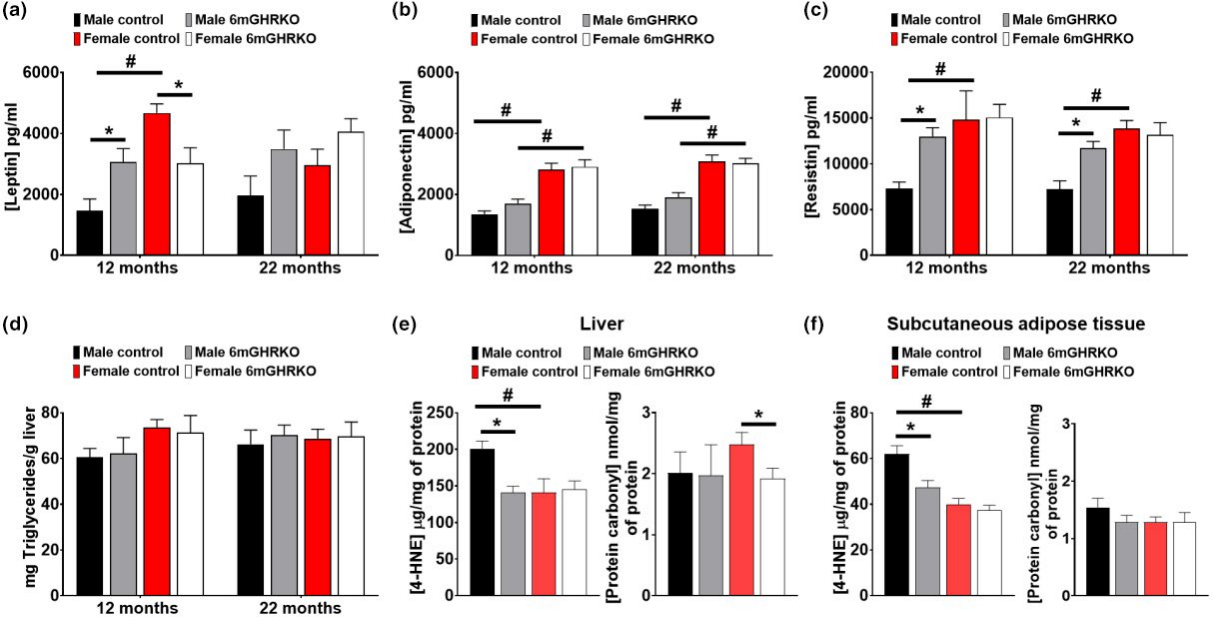
Increased adipokines and reduced oxidative damage in 6mGHRKO mice. (a) Circulating levels of leptin at 12 and 22 months of age (n = 9/group). (b) Circulating levels of adiponectin at 12 and 22 months of age (n = 9/group). (c) Circulating levels of resistin at 12 and 22 months of age (n = 9/group). (d) Liver triglycerides at 12 and 22 months of age (n = 7/group). (e‐f) HNE‐protein adducts and protein carbonyls measurements indicating lipid and protein peroxidation, respectively, in the subcutaneous adipose tissue and liver protein samples of male and female 12‐month‐old 6mGHRKO and control littermates under fasting conditions (n = 8–10). Black bars represent male and red bars female controls, whereas gray‐ and white bars represent male and female 6mGHRKO mice, respectively. Student two‐tailed T test was used to assess significant differences between experimental and control mice of the same sex and two‐way ANOVA was used to determine differences among sexes and groups. All values are mean ± SE. *p ≤ 0.05

As disruption of GH action in other mouse lines has been shown to decrease reproductive capabilities, we assessed the levels of the anterior pituitary FSH and LH and saw no change between 6mGHRKO mice and their respective controls (Figure S2). Additionally, both GH action and increased AT mass are associated with an altered adipokine expression (Kopchick et al., 2020), and lipid and protein oxidation can modulate many obesity‐related comorbidities (Manna & Jain, 2015). Thus, we assessed circulating levels of three adipokines: leptin, adiponectin and resistin, as well as the protein and lipid oxidation in the liver and subq AT. Consistent with the subq AT mass (Figure S3), which was only increased in male mice, serum leptin and resistin levels were significantly elevated in male 6mGHRKO mice at 12‐ and 22 months of age (*p* < 0.0141; Figure 4a,c), while serum leptin levels were significantly decreased in 6mGHRKO females at 12 months (*p* < 0.0124; Figure 4a). Adiponectin levels were unchanged between 6mGHRKO mice and controls, though two‐way ANOVA showed significantly increased adiponectin levels in females versus males at both time points tested (*p* = 0.0003, Figure 4b).

As increased aging is associated with increased levels of oxidative damage, we evaluated the levels of protein carbonyls and 4‐Hydroxynonenal (HNE)‐protein adducts as indicators of protein oxidation and lipid peroxidation, respectively. We observed that HNE‐adducts were significantly decreased in the liver (*p* = 0.0009; Figure 4e) and subq (*p* = 0.0075; Figure 4f) of male 6mGHRKO mice. Interestingly, while female 6mGHRKO mice did not show a significant decrease in HNE‐adducts in liver and subq AT compared to controls, a significant decrease in lipid peroxidation in both tissues was seen in females (*p* < 0.0400) compared to males (Figure 4e,f), irrespective of *Ghr* knockout status. Furthermore, protein carbonyls were decreased in the liver of female 6mGHRKO mice (*p* = 0.0479; Figure 4e) compared to controls. Because of the obesity present in 6mGHRKO mice, liver triglycerides and inflammatory markers were also measured and were unchanged (Figure 4d, Table S1).

### 2.5 Extended median and maximal lifespan in 6mGHRKO female mice

**FIGURE 5:**
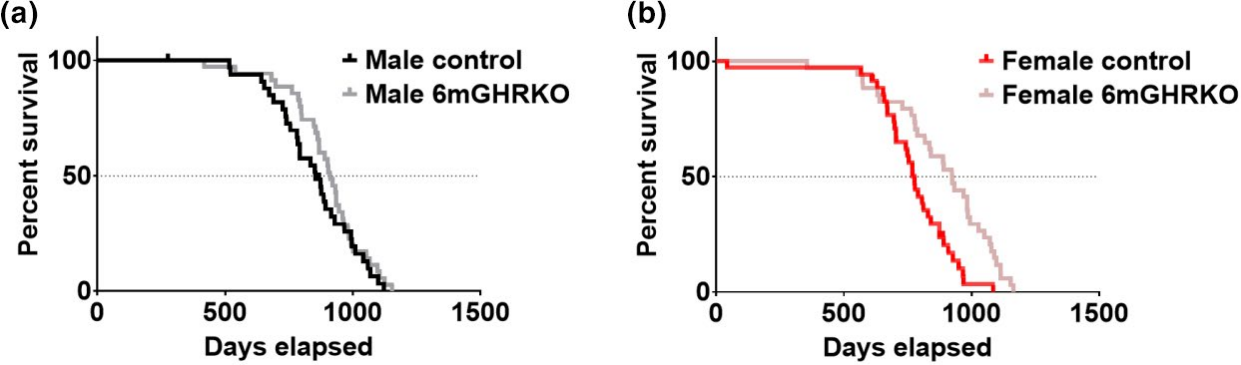
Disruption of GH action at a mature‐adult age extends lifespan in female mice. (a) Survival curve for males. (b) Survival curves for females. n = 32–36 mice per group. In both sexes, black and red curves represent male and female controls respectively, while gray and pink curves represent male and female 6mGHRKO mice, respectively. Females had a significant lifespan extension, Log‐rank (*p* = 0.00005) and Gehan‐Breslow‐Wilcoxon test (*p* = 0.0063). *p ≤ 0.05

Ablation of GH action in mice at 6 months of age significantly increased mean, median, and maximum lifespan in female mice. Mean lifespan was measured by both Log‐rank (*p* = 0.0007) and the Gehan‐Breslow‐Wilcoxon tests (*p* = 0.0063) (Figure 5b, Table S2). 6mGHRKO females showed a 20% increase in median survival, living 925 days compared to 769 days in female controls (*p* = 0.0178; Tables S3 and S4). Maximal lifespan in female 6mGHRKO mice was extended by 15%, with 6mGHRKO females living 1164 days compared to 1085 days of controls (*p* = 0.0278; Table S3). Although median survival for male 6mGHRKO mice was increased by 63 days vs controls (911 days vs. 852 days), it did not reach statistical significance (*p* = 0.0883) (Tables S3 and S4). Maximal lifespan in 6mGHRKO and control males was 1154 and 1122 days, respectively (*p* = 0.4338, Table S4).

### 2.6 Reduced neoplasms and glomerulonephritis in 6mGHRKO mice

**FIGURE 6:**
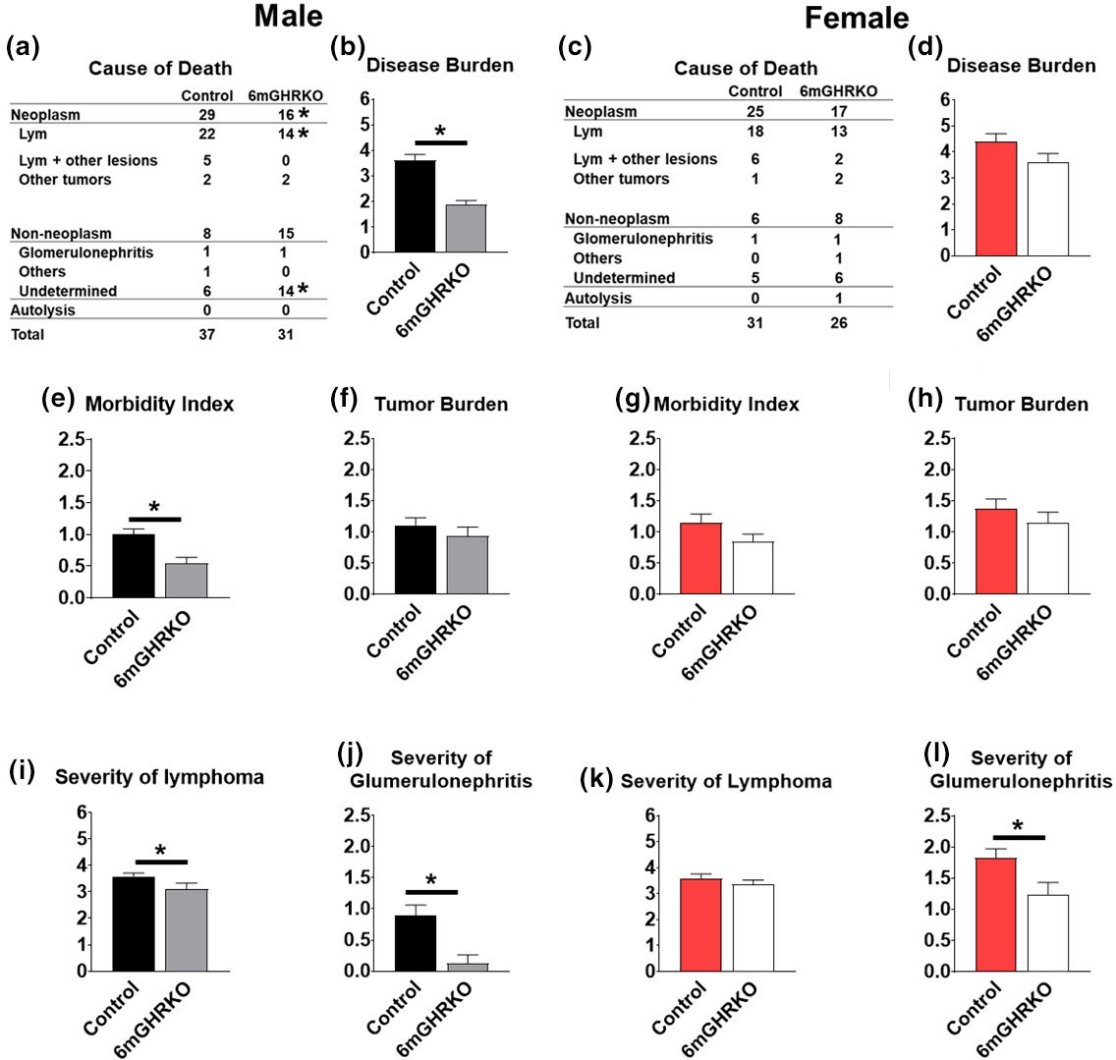
Reduced neoplasm in males but decreased severity of glomerulonephritis in male and female 6mGHRKO mice. (a and c) Probable cause of death in male and female mice. (b and d) Disease burden in male and female mice. (e and g) Morbidity index in male and female mice. (f and h) Tumor burden in male and female mice. (i and k) Severity of lymphoma in male and female mice. (j and l) Severity of glomerulonephritis in male and female mice. 6mGHRKO males. Black bars represent male and red bars represent female controls, whereas gray‐ and white bars represent male and female 6mGHRKO mice, respectively. Student two‐tailed T test was used to assess significant differences between experimental and control mice of the same sex. All values are mean ± SE. *p ≤ 0.05

Global congenital reduction in GH action (as seen in GHRKO mice and patients with LS) leads to resistance to cancer (Guevara‐Aguirre et al., 2011; Ikeno et al., 2009). Therefore, we performed end of life pathology analysis that revealed a significantly reduced incidence of fatal neoplastic lesions in male 6mGHRKO mice compared to the controls (*p* = 0.0201; Figure 6a), despite a comparable tumor burden in both sexes compared to controls (Figure 6f,h). Lymphoma was the most frequently fatal neoplastic occurrence in both sexes (Figure 6a,c); however, only male 6mGHRKO had a significantly lower severity of lymphoma compared to the control mice (*p* = 0.041; Figure 6i,f,k). Interestingly, the total disease burden, including neoplastic and non‐neoplastic diseases, and the morbidity index, which reflect age‐related accumulation of tissue and cell injury, were markedly lower for male 6mGHRKO (*p* < 0.0100; Figure 6b,e) but not in female (Figure 6d,g) 6mGHRKO mice compared to controls. Further, both male and female 6mGHRKO mice displayed a markedly reduced severity of glomerulonephritis (*p* < 0.0200; Figure 6j,l), consistent with prior observations in long‐lived congenital GH‐deficient Ames (Ikeno et al., 2003) and GH‐resistant GHRKO mice (Ikeno et al., 2009). Importantly, the cause of death in the 6mGHRKO male mice was categorized as ‘undetermined’ (45% or 14/31 cases in 6mGHRKO mice compared to 16% or 6/37 in male controls) by the terminal pathology analyses (Figure 6a).

## 3 DISCUSSION

Germline disruption of the GH/IGF‐1 axis such as seen in Ames, Snell, lit/lit, GHRKO, and GH knockout (GH−/−) mice leads to health and lifespan benefits (Bartke, 2008). In the present study, we tested how adult‐onset reductions in GH action affect health and lifespan, using a mouse line of inducible ablation of the GHR starting at 6 months of age (6mGHRKO). We found sexual dimorphic effects of 6mGHRKO on lifespan, glucose metabolism, and cancer. Together, the results suggest that decreased GH action via *Ghr* gene disruption in adult mice has a positive impact in terms of healthy lifespan.

An important finding of this study was the significantly extended longevity seen in female 6mGHRKO mice, with mean, median, and maximal lifespan increased compared to controls. Additionally, although increased lifespan was not seen in males, median lifespan extension nearly reached significance in male 6mGHRKO. Notably, median lifespan in 6mGHRKO female and males was increased by 20% (769 vs. 925 days) and 7% (852 vs. 911 days), respectively, while median lifespan in germline female and male GHRKO mice in the C57BL/6J background was increased by 20% (850 vs. 1023 days) and ~9% (866 vs. 941 days), respectively. Maximal lifespan assessment has not been reported for female GHRKO mice as at the time of the assessment some females were still alive (Coschigano et al., 2003). Interestingly, similar to 6mGHRKO mice, a sex‐specific longevity extension has also been reported in the 1.5mGHRKO mice (Junnila et al., 2016) albeit to a lesser degree—with only an extension in maximal lifespan, but no change in mean or median longevity in the female mice versus controls. Postnatal disruption of the mouse GH/IGF‐1 axis has been modeled in other additional aging studies: (1) liver–specific IGF‐1 deficient mice, with hepatic IGF‐1 ablation at three developmental stages, 10‐days (LID10d), 5 months (LID5m) and 15 months of age (LID15m) (Ashpole et al., 2017); (2) late life targeted IGF‐1 receptor (R) mice, treated with IGF‐1R monoclonal antibody (L2‐Cmu) onwards from 18‐months of age (Mao et al., 2018); and (3) pregnancy‐associated plasma protein‐A (fPAPP‐A/pos) mice, with the PAPP‐A gene ablated at 5 months of age (Bale et al., 2017). Interestingly, and in agreement to the results seen in 1.5mGHRKO and 6mGHRKO mice, extended mean, median or maximal longevity in these four studies were seen in females but not in males, suggesting that postnatal disruption of GH/IGF‐1 axis preferentially extends lifespan in females. Of significance, fPAPP‐A/pos mouse studies were made only in female mice due to possible detrimental Tam effects in male mice, such as scrotal enlargement and herniation (Bale et al., 2017). Because some reports indicate that Tam may have detrimental effects on male and female physiology, it is possible that the lack of lifespan extension seen in male 1.5mGHRKO and 6mGHRKO mice is influenced by Tam treatment. However, recent experimental evidence suggests otherwise. Donocoff et al. found that serum and histological changes driven by Tam injection in mice are reversed by 28 days post treatment (Donocoff et al., 2020). Furthermore, from the mice with postnatal disruption in the GH/IGF‐1 axis described above, only the fPAPP‐A/pos mice use Tam to drive gene disruption. Thus, the female specific advantage seen in these mice seems to be independent of Tam treatment, but dependent on the postnatal reduction of GH and/or IGF‐1 action.

Sexual dimorphism with respect to GH action has been documented before, for example, the liver expression of *CYP* genes (monooxygenase enzymes that metabolize diverse steroids and fatty acids) is sex‐specific and can be modified by adjusting the GH pulsatility (Ahluwalia et al., 2004). There is an interplay between the GH/IGF‐1 axis and sex hormones, as estrogen is well known to inhibit GH action (Leung et al., 2003). In fact, GH secretion pattern is sex‐dependent with females showing higher nadir GH levels than males, although the pulsatile excursions in males are more dramatic (Jansson et al., 1985; Leung et al., 2003). It also has been shown that estrogen via the estrogen receptor can interact and modulate activity of downstream effectors of the GHR signaling pathway, such as STAT5 (Leung et al., 2003). Furthermore, estrogen can inhibit JAK2 activation (which is required for GHR activation) by promoting the expression of Suppressor of cytokine signaling 2 (SOCS2) (Leung et al., 2003). To note, results released by the National Institute of Aging and the ‘Interventions Testing Program’ (ITP) have shown that postnatal dietary interventions that affect the IGF‐/insulin pathway such as Rapamycin have a greater effect in extending female longevity (Nadon et al., 2017). Although females with postnatal reduction in the GH/IGF‐1 axis have an advantage in terms of lifespan (Ashpole et al., 2017; Junnila et al., 2016; Mao et al., 2018), this sex‐specific advantage is yet to be clarified as mouse lines with germline decrease in GHR activation have lifespan extension in both, male and female mice (Bartke, 2008). Furthermore, studies performed with GH‐deficient Ames mice have shown that a 6‐week GH treatment starting at very young age (first or second week of age) have sexually dimorphic effects with respect to longevity, depending on the age at which the treatment is started. For example, GH treatment starting at 1‐week of age reduced the lifespan to that of control mice only in Ames males while starting GH treatment at 2‐weeks of age affected both sexes similarly (Sun et al., 2017). These studies suggest that there may be an important age‐window in which reduction of GH action modulates a sex‐specific lifespan advantage.

To evaluate the ablation of the *Ghr* gene in 6mGHRKO mice and the effects that this disruption has on the GH/IGF‐1 axis, we evaluated gene expression of *Ghr and Igf‐1* in several tissues and assayed the circulatory levels of GH, IGF‐1 and IGFBP‐2 and IGFBP‐3. We found that *Ghr* gene disruption was not uniform among all tissues. That is, while *Ghr* mRNA was more than ~50% reduced in all tissues tested, *Ghr* gene knockdown was more robust in liver and white AT than in skeletal and heart muscles. Also, because IGF‐1 expression is mostly regulated by GH signaling, it was expected that *Igf‐1* gene expression followed the *Ghr* expression pattern. Approximately 75%–90% of the circulating IGF‐1 is secreted by the liver (List et al., 2014) and because GH ablation was best in liver, we predicted and confirmed that similar to GHRKO mice, circulating IGF‐1 levels were significantly reduced. Interestingly, the serum levels of IGF‐1 were higher in female vs. male 6mGHRKO. This is congruent with reports showing that after 4 weeks of age there is a decrease in IGF‐1 levels in male but not female mice (Walz et al., 2020). Furthermore, Tam treatment may also have an effect in IGF‐1 serum levels as it has been shown that oral administration of estrogen and Tam in woman decreases IGF‐1 levels (Ho & Weissberger, 1992; Mandalà et al., 2001), while transdermal administration of estrogen elevates IGF‐1 levels (Ho & Weissberger, 1992). Therefore, the i.p. Tam injection may have the same effect as transdermal administration in increasing IGF‐1 levels in female 6mGHRKO mice. IGF‐1 binds to six IGFBPs. Therefore, IGFBPs impact circulating IGF‐1 bioavailability, half‐life, and activity (Juul, 2003). Furthermore, IGFBP‐2 is known to be suppressed by GH, insulin, and obesity and has been positively correlated with insulin sensitivity (Juul, 2003). Serum IGFBP‐3 is upregulated by GH and is the most abundant serum IGFBP (Juul, 2003; Rajaram et al., 1997). We found that the 6mGHRKO mice had increased IGFBP‐2 in males and decreased IGFBP‐3 levels in males and females. Of importance, ~95% of circulatory IGF‐1 is bound and forms a tertiary complex with IGFBP‐3 and a protein called acid‐labile subunit (ALS), which is also upregulated by GH (Juul, 2003). Therefore, due to the decreased IGFBP‐3 it is possible that more unbound IGF‐1 is found in circulation of 6mGHRKO mice, although confirmatory assays would have to be performed to evaluate the status of free circulatory IGF‐1. The alteration found in the GH/IGF‐1 axis were expectedly similar to GHRKO and 1.5mGHRKO mice (Coschigano et al., 2000; Junnila et al., 2016). One limitation of this study is that due to the lack of specificity of the GHR antibodies tested, the reduced GHR protein levels were not shown. Despite this, the phenotype of the 6mGHRKO mice is consistent with reduced GHR protein in the tissues, that is, the aforementioned changes in serum GH and IGF‐1 levels, and the increased adipose tissue and decreased lean mass compared to controls. Therefore, altogether these results confirm that the 6mGHRKO mice have reduced GH action, which, in turn, modulates the IGF system.

Growth hormone has a diabetogenic effect, reducing insulin signaling (Jansson et al., 1985), and therefore, one mechanism that has been associated with lifespan extension is improved glucose homeostasis (Templeman et al., 2017). Long lived mice with decreased GH/IGF‐1 signaling, such as Ames, Snell, GHRKO, GH knockout (GH−/−), and 1.5mGHRKO mice, have consistently shown improved insulin sensitivity (Junnila et al., 2016). We found that 6mGHRKO mice had a sex‐specific effect with respect to insulin sensitivity, wherein males showed improved while females had intact insulin sensitivity compared to controls. Despite the unchanged ITT in female 6mGHRKO vs controls, increased adiponectin levels (an adipokine associated with insulin sensitivity) and decreased fasting glucose levels were found in females (with or without Tam injection) compared to male mice. Also, glucose tolerance, fasting glucose and fasting insulin level were unaltered in both sexes of the 6mGHRKO mice. These results are in conflict with results obtained in Ames, germline GHRKO, or 1.5mGHRKO mice, which show reduced fasting insulin and glucose serum levels and decreased glucose tolerance (Bartke et al., 1998; Junnila et al., 2016). The discrepancy in glucose homeostasis findings indicate that the age at which GH action is diminished can have profound effects on glucose metabolism, as GH may affect glucose‐responsive organs differently in an age‐dependent manner. Glucose intolerance reported in mouse lines with germline disruption of GH action, such as in GHRKO and GH−/− mice, has been attributed to a possible decrease in insulin secretion due to smaller pancreatic islet size (List et al., 2019; Liu et al., 2004). In line with this, the pancreas weight of male and female 6mGHRKO mice was also reduced at 12 months of age. Thus, it is possible that the islet cells are also smaller. Kineman's group at the University of Illinois‐Chicago have reported on adult‐onset GH‐deficient mice (AOiGHD) with ~50% decreased circulating GH due to a postnatal (10 weeks of age) targeted destruction of the somatotrophs. Studies performed on AOiGHD and beta cell‐specific‐GHRKO mice show unchanged beta cell mass with altered insulin response, suggesting that other factors such as lipotoxicity can affect insulin response (Cordoba‐Chacon et al., 2014; Luque et al., 2011). Along the same line and similar to germline GHRKO and male 1.5mGHRKO mice (Berryman et al., 2010; Junnila et al., 2016), 6mGHRKO mice have an obese phenotype but did not show any changes in liver TG in both sexes at both time points. These results contrast with the decreased liver TG found in AOiGHD mice and female 1.5mGHRKO mice (Junnila et al., 2016; Luque et al., 2011). We did not test the mechanisms that affect glucose homeostasis such as lipotoxicity and difference in glucose uptake capacity, but due to the altered body composition seen in 6mGHRKO mice, investigating such mechanisms by means of evaluating circulating triglycerides, free fatty acids, and glucose uptake in individual tissues is of interest and warrants future studies.

Possible therapeutic interventions to extend healthy aging are more clinically relevant at an adult age and it has been shown that early life disruption in GH action affects not only longevity but also longitudinal growth and body composition. We also evaluated these parameters in 6mGHRKO mice and found that unlike the 1.5mGHRKO mice, both male and female 6mGHRKO mice had no significant changes in bodyweight and minimal impact in body length (Junnila et al., 2016). Despite these results, the relative size of some internal organs was changed in 6mGHRKO mice in a sex‐ and age‐specific manner, suggesting that GH not only supports growth, but also has a role in the maintenance of many internal organs. As mentioned above, GH has a role in body composition. It has been shown that GH has a catabolic effect on AT, increasing lipolysis and decreasing lipogenesis; consequently, reduced GH action leads to AT enlargement (Kopchick et al., 2020). Since obesity is commonly correlated with diseases such as cancer, diabetes and cardiovascular diseases (Garg et al., 2014), the “obese, but healthy” phenotype seen in GHRKO, GH−/−, 1.5mGHRKO and the AOiGHD mice has drawn the attention of many investigators to understand how the “quality and not the quantity” of AT impacts health and longevity; It was suggested that some AT depots have more protective effects than others (Troike et al., 2017). As such, the subq AT has been associated with healthy obesity than the visceral depots as the preferential distribution of excess lipids in the subq depot is known to be less detrimental than at the visceral depots (Troike et al., 2017). In fact, it has been shown that in obese rats, transplantation of autologous subq AT into two visceral depots decreases insulin resistance (Torres‐Villalobos et al., 2016). Furthermore, hepatic insulin resistance can be reversed by removing visceral fat (Barzilai et al., 1999). Of importance, mice with reduced GH action (GHRKO, GH−/−, GHA) and mice with adipocyte‐specific reduction in GH action (AdGHRKO), have a preferential enlargement in the subq depot with relatively little alteration to the perigonadal depot, suggesting that subq and peri are the most and the least GH impacted AT depots, respectively (Berryman et al., 2010). Likewise, characteristics such as adipocyte size, fibrosis, and immune cell infiltration appear to be more robustly altered in the subq AT depot of GHRKO, GH−/−, and AdGHRKO mice (List et al., 2019; List et al., 2019). Accordingly, the 1.5mGHRKO and the 6mGHRKO showed a greater enlargement in the subq depot while the AT mass of the peri and retro depots were the least altered (Junnila et al., 2016). Interestingly, the lipid and protein oxidation 6mGHRKO mice seem to be reduced in the subq and liver tissues of 6mGHRKO mice in a sex‐dependent manner further suggesting that the subq AT enlargement seen in 6mGHRKO mice may have a positive “healthy impact” in the phenotype.

One of the postulated mechanisms for extending lifespan in states of decreased GH action is resistance to cancer as both GHRKO and Ames mice, as well as patients with LS showed reduced incidence of cancer. Accordingly, male 6mGHRKO mice showed lower cancer incidence compared to controls. Although this may appear counterintuitive with respect to the longevity results, it is important to note that the post‐mortem pathology analyses were conducted at the end of life. Thus, the female 6mGHRKO samples were significantly older than the post‐mortem specimens for control female mice and had more time to develop cancer than control mice (Ikeno et al., 2003). In light of this possibility, it would be interesting to be able to evaluate age‐matched cohorts for intermittent pathology to observe more precise causes of death across the sexes and ages. Furthermore, other mouse lines with adult‐onset reduction in the GH/IGF‐1 axis also have shown controversial results with respect to cancer resistance as lifespan extension seen in LID5m females was not associated with reduced cancer incidence (Ashpole et al., 2017). Conversely, female L2‐Cmu treated mice show reduced deaths due to cancer and compared to controls (Mao et al., 2018). Another potential reason why female mice did not show notable effects on pathology is the sexual dimorphism with respect to the spectrum of cancer. For example, female mice have more pituitary tumors than male mice (Seldon et al., 1996). Since this was the post‐mortem pathological assessment, there was degradation of the tissues at various degree and the central nervous system (including pituitary gland) is one of the organs that deteriorates faster than others; thus, there is a possibility that the changes in incidence and/or severity of pituitary adenoma could not be detected. Importantly, even though adult‐onset disruption of GH axis seems to improve female lifespan, health span enhancement is still controversial as LID and L2‐Cmu mice do not show any improvements in glucose homeostasis (Mao et al., 2018), and female LID mice show decreased physical and cognitive performance, as well as no difference in cancer induced mortality when compared to controls (Ashpole et al., 2017). RNA sequencing experiments, frailty tests, rotarod, and grip strength studies that are currently ongoing will help to more adequately elucidate the healthspan and possible cellular mechanisms altered in 6mGHRKO mice.

In summary, we generated a mouse line with ablated GHR at a mature‐adult age of 6 months (6mGHRKO). We found that 6mGHRKO mice did not have decreased body size and were GH‐resistant (low IGF‐1 and high GH circulatory levels), with decreased lean mass and increased adiposity, especially in the subq depot. Despite the obese phenotype, 6mGHRKO mice showed normal liver TGs and reduced oxidative damage. Importantly, females exhibited significant mean, median, and maximal lifespan extension while improved insulin sensitivity, cancer resistance, and a trend to extend median lifespan was seen in males. Hence, we show that suppressing GH action at a mature‐adult age results in normal pubertal growth and confers multiple benefits to long‐term health.

## 4 EXPERIMENTAL PROCEDURES

### 4.1 Generation and maintenance of 6mGHRKO mice

Mice with a C57BL/6J genetic background carrying Lox P sites flanking exon 4 of the *Ghr* gene were previously produced by the Knockout Mouse Project (KOMP) (Junnila et al., 2016). C57BL/6J mice expressing a ubiquitous Cre recombinase gene driven by the ROSA26 gene promoter/enhancer (B6.129‐Gt(ROSA)26Sortm1(cre/ERT2)Tyj/J mice) were purchased from The Jackson Laboratory (Duran‐Ortiz et al., 2018; Junnila et al., 2016). Mice were bred to homozygosity for both the floxed *Ghr* gene and the Cre recombinase gene. To ablate the *Ghr* gene, 6‐month‐old mice received intraperitoneal injections of 95–110 µl of Tam dissolved in peanut oil (6mGHRKO mice) or vehicle (peanut oil) to control mice. A total dose of 0.32 mg of Tam/g of bodyweight was administered; mice received an injection once per day over 5 consecutive days, as described previously (Duran‐Ortiz et al., 2018). Three cohorts of male and female mice were used as follows: two experimental groups to perform the phenotypic and metabolic characterization and a cohort for longevity studies. The first experimental cohort was dissected at middle age or 12 months old (n = 12); the second experimental cohort was dissected at an old age or 22 months old (n = 12). The longevity study cohort were followed throughout their lives and the time of death recorded (n = 30–35) with no other experimental manipulation. Mice were housed in a temperature and humidity‐controlled room at 22°C under a 14‐h light, 10‐h dark cycle, with 3–4 mice per cage, and with ad libitum access to water and standard laboratory chow (ProLab RMH 3000). All mouse protocols were approved by Ohio University's Animal Use and Care Committee.

### 4.2 Serum collection and tissue dissection

Serum was collected from blood obtained from the orbital sinus. Dissections of the experimental groups took place after overnight fasting. Euthanasia was performed using CO_2_ and cervical dislocation. After euthanasia, mice were dissected, and organs harvested. Collected organs were weighed, snap frozen in liquid nitrogen and stored at −80°C.

### 4.3 Validation of the global GHR gene disruption at 6 months of age

To validate GHR disruption, RT‐qPCR was used to evaluate *Ghr* and *Igf‐1* gene expression in the indicated tissues: liver, subq AT, peri AT, kidney, quadriceps (quad) skeletal muscle, and heart. Experiments to assess the circulating levels of GH and IGF‐1 were also performed using ELISA as described below.

#### 4.3.1 RT‐qPCR

For RNA isolation, frozen tissues were homogenized using a Precellys 24‐Dual homogenizer. RNA was isolated using the Thermo Scientific™ GeneJET RNA Purification Kit following manufacturer's instructions. The quantity and quality of total RNA was measured with the NanoDrop ND‐2000 (Thermo Scientific). To ensure purity and quality of the RNA, only samples with a 260/280 and a 260/230 ratio ≥1.8 were used for subsequent experiments. cDNA synthesis was performed using the Maxima First Strand cDNA Synthesis Kit for RT‐quantitative PCR (RT‐qPCR), and Maxima SYBR Green/Fluorescein qPCR Master Mix (Thermo Scientific) was utilized to perform the RT‐qPCR. Samples were quantified using a Bio‐Rad iCycler (Bio‐Rad Laboratories). Two housekeeping genes were used to assess Ghr and Igf‐1 gene expression. RT‐qPCR data analysis was performed using qBasePlus software (Biogazelle—www.qbaseplus.com), which allows the normalization to more than one reference gene, as well as correction for primer efficiency and between plate replication. Primers used are indicated in Table S4.

#### 4.3.2 ELISA for GH and IGF‐1 measurements

Serum collected at the time of dissection was used to measure GH and IGF‐1. The mouse/rat‐GH (22‐GHOMS‐E01) and the mouse/rat IGF‐1 (22‐IG1MS‐E01) ELISA Kits from ALPCO were used following manufacturer's instructions.

### 4.4 Body composition and length

A Bruker Minispec NMR analyzer (Bruker Corp.) was used to measure body composition, which was determined every month starting 1 day before Tam treatment until the time of dissection (12 or 22 months of age), as previously described (Junnila et al., 2016). Body length was measured at the time of dissection from the tip of the nose to the anus. Femur length was measured using a digital caliper.

### 4.5 Glucose metabolism

Insulin tolerance test (ITT) and a glucose tolerance test (GTT) were performed at 11 months and 21 months of age, as previously described (List et al., 2014). For GTTs, a 10% glucose solution was prepared in filtered PBS. GTT was performed in overnight fasted mice injected ip. with 0.01‐ml glucose solution/g bodyweight. ITT was performed in 6 h fasted mice, injected ip. with recombinant human insulin (Novolin‐R; Novo Nordisk), 0.01‐ml/g bodyweight out of 0.075 U/ml stock solution. Blood glucose was measured before injections and 15, 30, 45, 60, and 90 min after injection. Since 21 months old males were more insulin resistant than females, we used 1.5 U/ml of insulin for ITT at this time point. All glucose measurements were taken using OneTouch Ultra glucose strips and glucometers (Lifescan).

### 4.6 Liver triglycerides

Liver triglycerides were evaluated using the frozen livers from mice at 12 and 22 months of age, as previously described (Salmon & Flatt, 1985). Briefly, 50 to 100‐mg of frozen livers collected from dissection were thawed and digested for 1 h at 37°C in 3 M KOH/65% ethanol and then neutralized with 2 M Tris HCl. Triglyceride content was determined using the triglycerides (GPO) reagent (T7532; Pointe Scientific) followed by spectrophotometric quantification (Salmon & Flatt, 1985).

### 4.7 Protein and lipid oxidation measurements

#### 4.7.1 Protein oxidation assay

The protein carbonyl derivatives of Pro, Arg, Lys, and Thr were measured in liver and subq AT lysates of the 6mGHRKO and control mice (n = 8–10), using the OxiSelect™ Protein Carbonyl ELISA Kit from Cell Biolabs following manufacturer's instructions.

#### 4.7.2 Lipid peroxidation

The byproduct of lipid peroxidation, 4‐hydroxynonenal (4‐HNE), can react with the lysine, histidine, or cysteine residues of proteins and form stable adducts. 4‐HNE‐adducts were measured in the protein lysates of liver and subq AT of 6mGHRKO and control mice (n = 8–10) using the OxiSelect™ HNE‐adduct competitive ELISA Kit from Cell Biolabs, following manufacturer's instructions. Absorbance of both ELISA assays were measured using Spectra Max 250 spectrophotometer at 450 nm.

### 4.8 Blood parameters

Insulin, leptin, resistin, interleukin‐6 (IL‐6), and monocyte chemoattractant protein‐1 (MCP‐1), as well as insulin‐like growth factor‐binding proteins (IGFBP)‐2 and −3 were determine in serum using MILLIPLEX MAP Mouse Metabolic Hormone Magnetic Bead Panel kit (MMHMAG‐44K) on a Milliplex 200 Analyzer (Millipore), following manufacturer's instructions.

### 4.9 Histopathology

End of life histopathology was performed in male 6mGHRKO (n = 31) and control (n = 37) mice, as well as female 6mGHRKO (n = 26) and control (n = 31) mice. At the end of life, mice were preserved in 10% formalin and shipped to the University of Texas at San Antonio Pathology Core. Tissues were infiltrated with paraffin and H&E sections were obtained. Slides were evaluated by two pathologists who were blinded to the experimental. Diagnosis of each histopathological change was made using histological classifications for aging mice in which a list of lesions was compiled for each mouse that included both neoplastic and non‐neoplastic diseases. Based on these histopathological data, tumor burden, disease burden, morbidity index, and severity of lesions in each mouse were assessed. Tumor burden was calculated as the sum of the different types of tumors in each mouse. The disease burden was calculated as the sum of the histopathological changes in a mouse and severity of neoplastic and renal lesions was assessed using an established grading system. In cases with neoplastic lesions, mice with Grade 3 or 4 lesions were categorized as death by neoplastic disease. In more than 90% of cases, there was agreement by the two pathologists. In cases where the two pathologists did not agree or where disease did not appear severe enough, the cause of death was categorized as unknown.

### 4.10 Statistics

Statistical analyses were performed using GraphPad Prism version 5.01 (GraphPad Software). All values are reported as mean ± SE. Student two‐tailed T tests were used to assess differences between 6mGHRKO mice vs. controls within a sex. These measurements included serum parameters, body length, organ sizes, liver triglyceride, and individual time points in GTTs, and ITTs. Two‐way ANOVA was used to evaluate differences between sexes and between experimental groups. Repeated measures two‐way ANOVA was used for bodyweight, fat mass, percentage of fat mass, lean mass, and percentage of lean mass over time were. A log‐rank test and a Gehan‐Breslow‐Wilcoxon test were used for comparison of survival data. For maximal and median lifespan comparisons, Fisher's exact test was used on contingency tables of the animals above the 90th and 50th age percentile, respectively (Han et al., 2016). The significance level of all experiments is set at *p* < 0.05. Survival analysis was performed using OASIS 2 (Online Application for the Survival Analysis 2) (Han et al., 2016).

## CONFLICT OF INTEREST

The authors declare no conflict of interests.

## AUTHOR CONTRIBUTIONS

Study conception and design: SDO, JJK. Acquisition of data: SDO, YI, JY, SB, TM, KF, SM, YQ, PK, SY. Analysis and interpretation of data: SDO, YI, RB, DB, EL. Manuscript preparation: SDO, JJK, DB, EL.

## Supporting information

Supplementary Material

## Data Availability

The datasets generated and analyzed during the current study are available upon request. Our studies do not include the use of custom code or mathematical algorithms.
