# Supplementary material for: Growth hormone receptor gene disruption in mature‐adult mice improves male insulin sensitivity and extends female lifespan
Source: Aging Cell. 2021 Nov 22;20(12):e13506. doi: 10.1111/acel.13506 (PMC8672790; doi:10.1111/acel.13506)
Supplement: Supplementary file 1 — Supplementary Material [file ACEL-20-e13506-s001.docx]

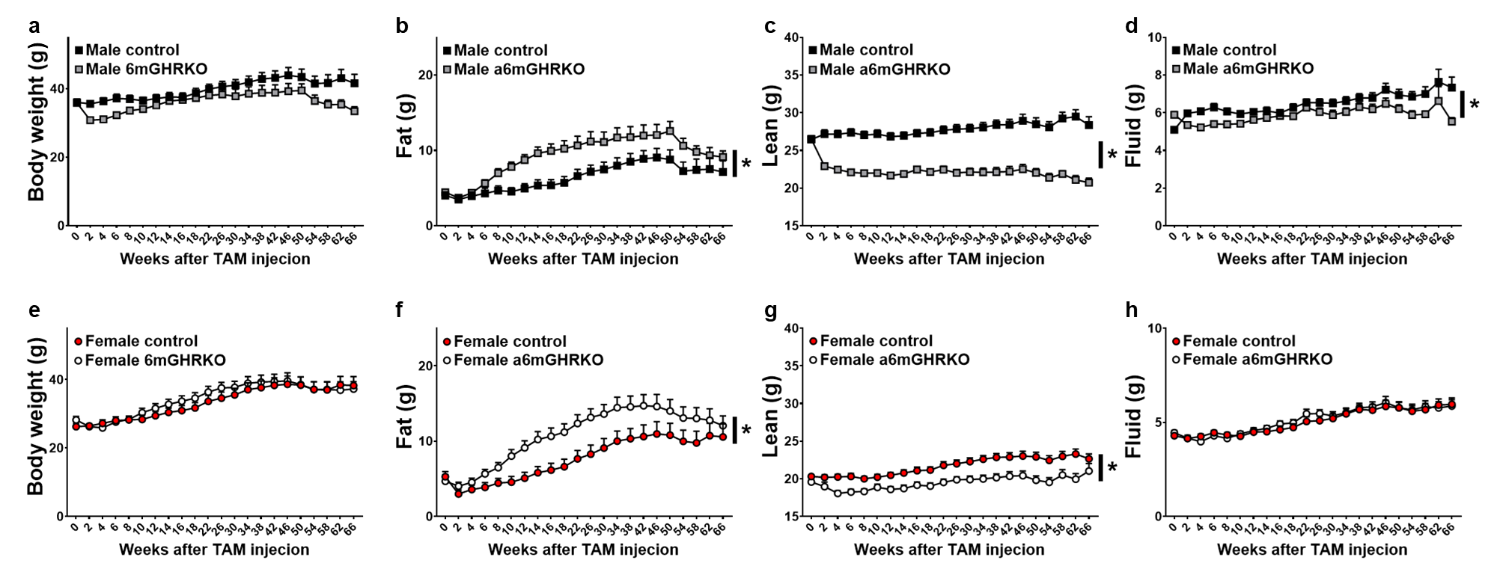


**Supplemental Figure 1.**

**
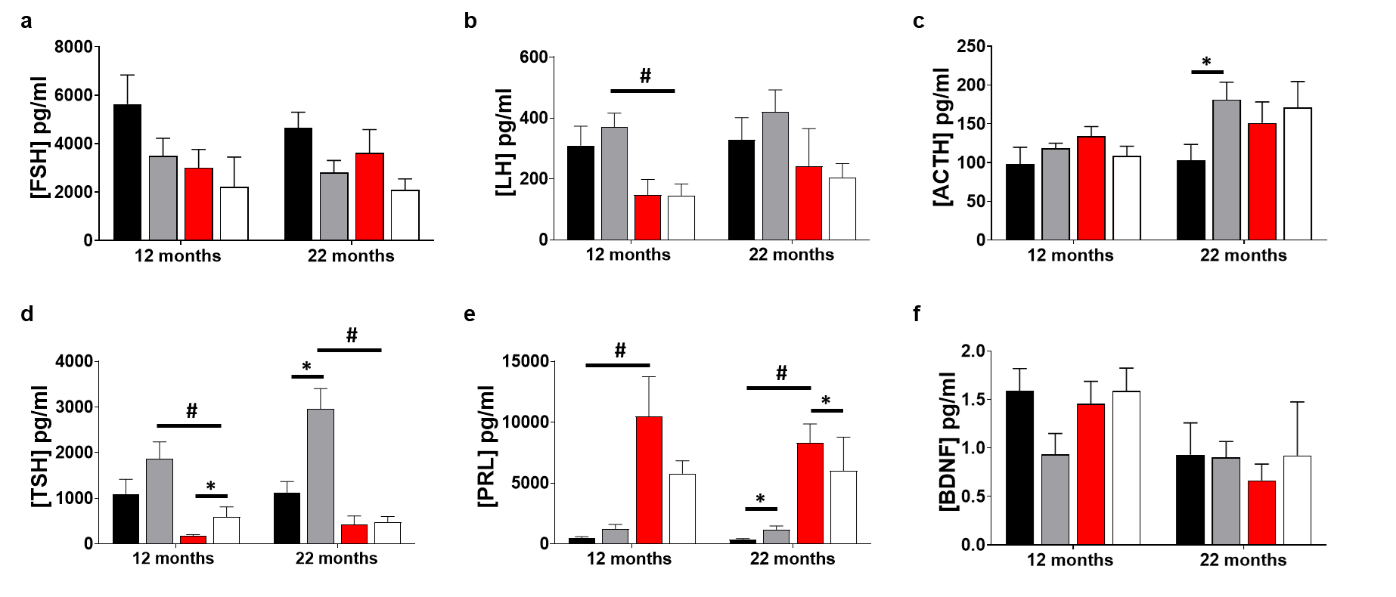
**

**Supplemental Figure 2.**


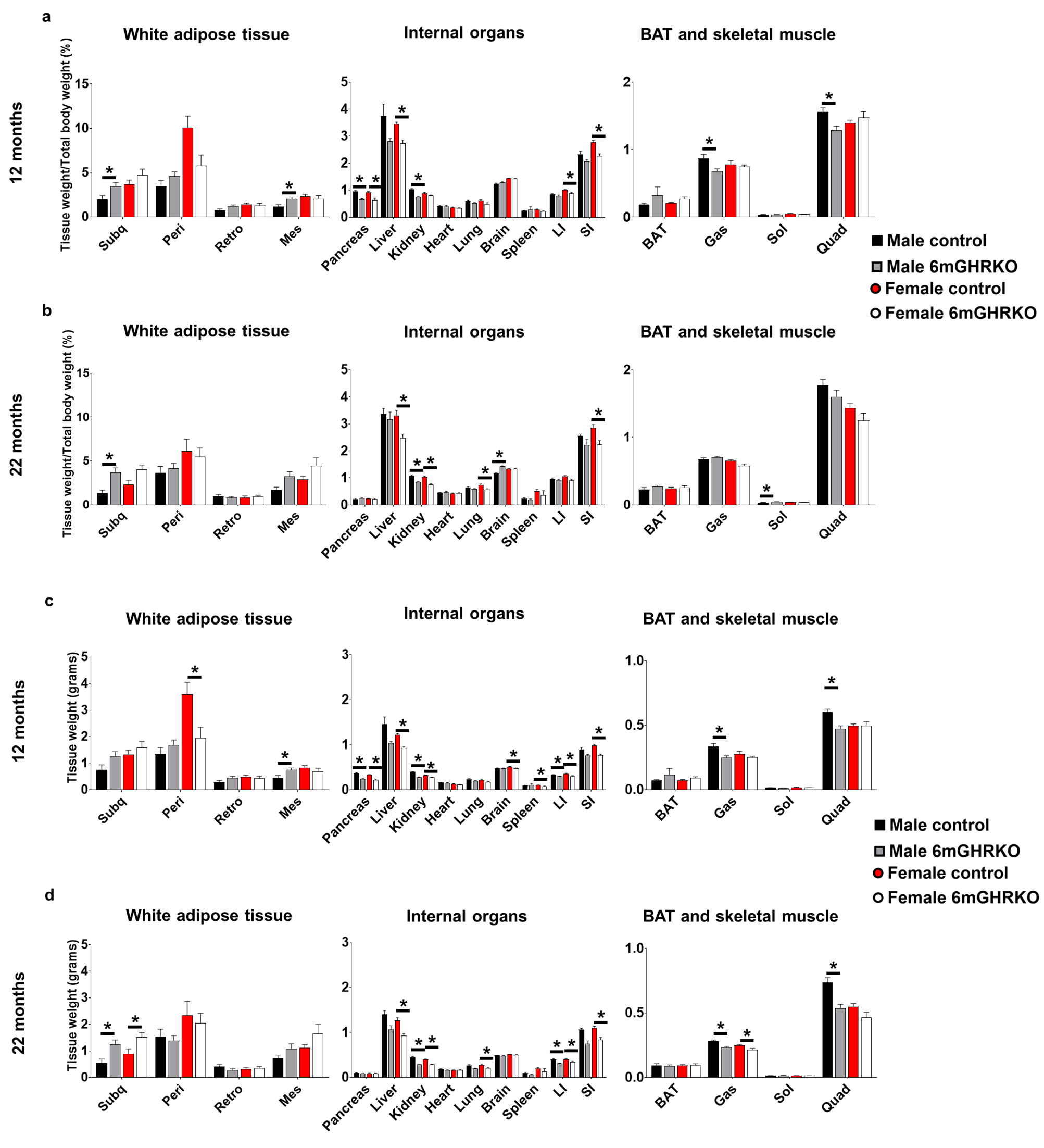


**Supplemental Figure 3.**

**
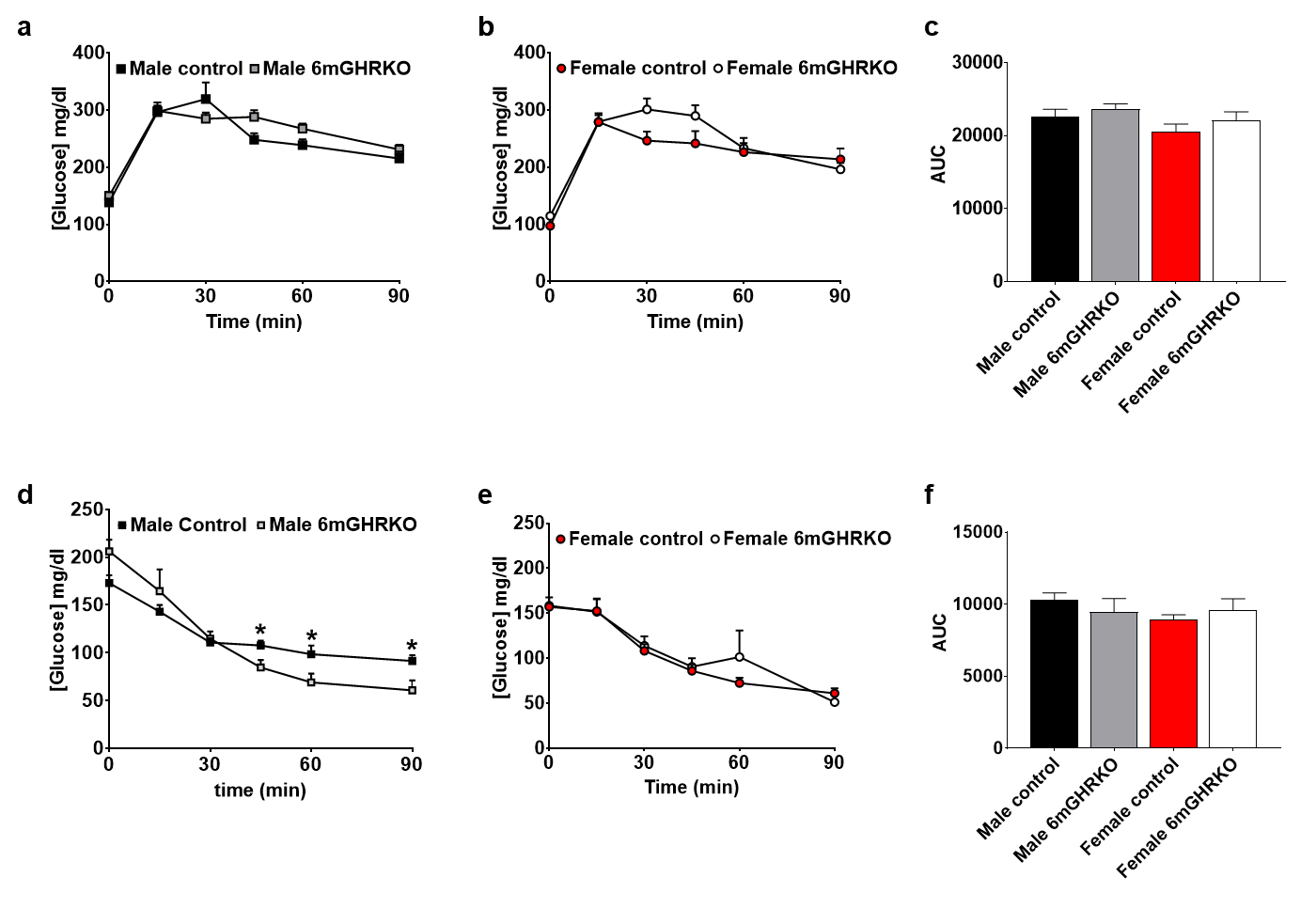
**

**Supplemental Figure 4.**

**Supplemental Table 1. No change in inflammatory profile in 6mGHRKO mice.**

|  |  | **Male control** | | **Male 6mGHRKO** | | **Female control** | | **Female 6mGHRKO** | |
| --- | --- | --- | --- | --- | --- | --- | --- | --- | --- |
|  |  | Mean | SE | Mean | SE | Mean | SE | Mean | SE |
| **MCP-1** | | 75.3 | 16.8 | 264 | 90.1 | 41.8 | 14 | 137 | 46.7 |
| **IL-6** | | 25.06***** | 9.23 | 93.3 | 19.4 | 188 | 115 | 63.1 | 17.3 |
| **TNF-a** | | 12.9 | 3.79 | 56.1 | 33 | 11.5 | 4.31 | 27.8 | 12 |

Circulating levels of Interleukin-6 (IL-6), macrophage chemoattractant protein -1 (MCP-1) and tumor necrosis factor α (TNF-α) in 6mGHRKO mice at 22 months of age (n=9/group). Student T test was used to assess significant differences between experimental and control mice of the same sex. All values are mean ± SE. * P ≤ 0.05.

**Supplemental Table 2. Disruption of GHR at 6 months of age extends mean lifespan.**

| **Log-Rank Test** | | | |
| --- | --- | --- | --- |
| **Condition** | **χ2** | **P-value** | **Bonferroni P-value** |
| Male control v.s. Male 6mGHRKO | 2.43 | 0.1193 | 0.358 |
| Male control v.s. Female control | 3.03 | 0.0816 | 0.2448 |
| Male control v.s. Female 6mGHRKO | 3.3 | 0.0693 | 0.2078 |
| Male 6mGHRKO v.s. Male control | 2.43 | 0.1193 | 0.358 |
| Male 6mGHRKO v.s. Female control | 11.33 | 0.0008 | 0.0023 |
| Male 6mGHRKO v.s. Female 6mGHRKO | 0.32 | 0.5734 | 1 |
| Female control v.s. Female 6mGHRKO | 11.48 | 0.0007 | 0.0021 |
|  | | | |
| **Wilcoxon-Breslow-Gehan Test** | | | |
| Male control v.s. Male 6mGHRKO | 2.98 | 0.0845 | 0.2534 |
| Male control v.s. Female control | 3.08 | 0.0792 | 0.2377 |
| Male control v.s. Female 6mGHRKO | 1.72 | 0.19 | 0.5699 |
| Male 6mGHRKO v.s. Male control | 2.98 | 0.0845 | 0.2534 |
| Male 6mGHRKO v.s. Female control | 13.03 | 0.0003 | 0.0009 |
| Male 6mGHRKO v.s. Female 6mGHRKO | 0 | 0.9496 | 1 |
| Female control v.s. Male 6mGHRKO | 13.03 | 0.0003 | 0.0009 |

Lifespan of male and female 6mGHRKO and control mice was evaluated using both log-rank test and Wilcoxon test as the later gives more weight to deaths at early time points, while the log-rank test gives equal weight to all time points. * P ≤ 0.05.

**Supplemental Table 3. Disruption of GHR at 6 months of age extends median and maximal lifespan.**

| **Fisher exact test** | | | | |
| --- | --- | --- | --- | --- |
| **Condition** | **P-value at 25%** | **P-value at 50%** | **P-value at 75%** | **P-value at 90%** |
| **Male control v.s. Male 6mGHRKO** | 0.0477 | 0.0883 | 1 | 0.4338 |
| **Male control v.s. Female control** | 0.4006 | 0.3311 | 0.1036 | 0.2409 |
| **Male control v.s. Female 6mGHRKO** | 0.4006 | 0.0883 | 0.2719 | 0.4307 |
| **Male 6mGHRKO v.s. Male control** | 0.0477 | 0.0883 | 1 | 0.4338 |
| **Male 6mGHRKO v.s. Female control** | 0.013 | 0.002 | 0.0309 | 0.2603 |
| **Male 6mGHRKO v.s. Female 6mGHRKO** | 0.2848 | 0.6376 | 0.4304 | 0.4782 |
| **Female control v.s. Female 6mGHRKO** | 0.1727 | 0.0178 | 0.0004 | 0.0278 |

Median (at 50%) and maximal (at 90%) lifespan of male and female 6mGHRKO and control mice was evaluated Fisher exact test. * P ≤ 0.05.

**Supplemental table 4. Primer sequences (5’ to 3’) by Sigma-Aldrich. GeNorm was used to select the most stable primer combinations for reference genes.**

|  | **Forward primer** | **Reverse primer** |
| --- | --- | --- |
| ***Ghr*** | GCCTGGGGACAAGTTCTTCTGGA | TGCAGCTTGTCGTTGGCTTTCCC |
| ***Igf1*** | GAGACTGGAGATGTACTGTG | CTTCCTCTACTTGTGTTCTTC |
| ***Eef2*** | TCGGCGCGCTTCCCTGTTCAC | ATGCCAGCCTTGCACACAAGGG |
| ***Eps3*** | ATCAGAGAGTTGACCGCAGTT | AATGAACCGAAGCACACCATA |
| ***Hprt*** | ATCAGTCAACGGGGGACATA | AGAGGTCCTTTTCACCAGCA |
| ***B2m*** | CTGGTCTTTCTATATCCTGGCT | CATGTCTCGATCCCAGTAGAC |
| ***Actb*** | CAGCTTCTTTGCAGCTCCTT | CACGATGGAGGGGAATACAG |
| ***Rpl38*** | CGCGTCGCCATGCCTCGGAA | ACTTGGCATCCTTCCGCCGGG |
| ***Eif3f*** | TACGAACGCCGCAACGAGGG | TGGCACCGAAAAGCAGTTGGTGA |
